# PEG-Functionalized Magnetite Nanoparticles for Modulation of Microbial Biofilms on Voice Prosthesis

**DOI:** 10.3390/antibiotics11010039

**Published:** 2021-12-29

**Authors:** Mara Caciandone, Adelina-Gabriela Niculescu, Aurelian Radu Roșu, Valentina Grumezescu, Irina Negut, Alina Maria Holban, Ovidiu Oprea, Bogdan Ștefan Vasile, Alexandra Cătălina Bîrcă, Alexandru Mihai Grumezescu, Miruna Silvia Stan, Alina Georgiana Anghel, Ion Anghel

**Affiliations:** 1 “Carol Davila” University of Medicine and Pharmacy, 050474 Bucharest, Romania; mara.caciandone@yahoo.com (M.C.); dr_alina.anghel@yahoo.com (A.G.A.); ionangheldoc@yahoo.com (I.A.); 2 Department of Science and Engineering of Oxide Materials and Nanomaterials, Faculty of Applied Chemistry and Materials Science, Politehnica University of Bucharest, 011061 Bucharest, Romania; adelina.niculescu@upb.ro (A.-G.N.); radu_rosu94@yahoo.com (A.R.R.); bogdan.vasile@upb.ro (B.Ș.V.); ada_birca@yahoo.com (A.C.B.); 3 Lasers Department, National Institute for Lasers, Plasma and Radiation Physics, 077125 Magurele, Romania; valentina_grumezescu@yahoo.com (V.G.); negut.irina@inflpr.ro (I.N.); 4 Department of Microbiology and Immunology, Faculty of Biology, University of Bucharest, 077206 Bucharest, Romania; alina_m_h@yahoo.com; 5 Research Institute of the University of Bucharest—ICUB, University of Bucharest, 050657 Bucharest, Romania; 6 Department of Inorganic Chemistry, Physical Chemistry and Electrochemistry, Faculty of Applied Chemistry and Materials Science, Politehnica University of Bucharest, 011061 Bucharest, Romania; ovidiu.oprea@upb.ro; 7 Academy of Romanian Scientists, Ilfov No. 3, 050044 Bucharest, Romania; miruna.stan@bio.unibuc.ro; 8 Department of Biochemistry and Molecular Biology, Faculty of Biology, University of Bucharest, 050095 Bucharest, Romania; 9 ENT Department, Saint Mary Clinical Hospital Bucharest, 011172 Bucharest, Romania; 10 “Dr. Carol Davila” Central Military Emergency University Hospital, 010825 Bucharest, Romania

**Keywords:** magnetite nanoparticles, PEG, polymyxin B, matrix assisted pulsed laser evaporation, antimicrobial resistance, antimicrobial coatings, antibiofilm activity

## Abstract

This study reports the fabrication of nanostructured coatings based on magnetite, polyethyleneglycol, and biologically active molecule (polymyxin B-PM) for producing biofilm-resistant surfaces (voice prosthesis). Magnetite nanoparticles (MNPs) have been synthesized and functionalized using a co-precipitation method and were further deposited into thin coatings using the matrix-assisted pulsed laser evaporation (MAPLE) technique. The obtained nanoparticles and coatings were characterized by X-ray diffraction (XRD), thermogravimetric analysis with differential scanning calorimetry (TGA-DSC), scanning electron microscopy (SEM), transmission electron microscopy with selected area electron diffraction (TEM-SAED), Fourier-transform infrared spectroscopy (FT-IR), and infrared microscopy (IRM). Their antibiofilm activity was tested against relevant *Staphylococcus aureus* and *Pseudomonas aeruginosa* bacterial strains. The Fe_3_O_4_@PEG/PM surface of modified voice prosthesis sections reduced the number of CFU/mL up to four orders of magnitude in the case of *S. aureus* biofilm. A more significant inhibitory effect is noticed in the case of *P. aeruginosa* up to five folds. These results highlight the importance of new Fe_3_O_4_@PEG/PM in the biomedical field.

## 1. Introduction

Antibiotics have undoubtedly revolutionized medicine in the past century, being used in treating and preventing the infections produced by bacterial strains [1,2,3,4]. In time, numerous such antimicrobials have been implemented for suppressing and killing pathogens, significantly reducing the occurrence of infectious diseases [4]. Nonetheless, the overuse and/or misuse of antibiotics was seen to cause mutations of the microorganisms that endowed them with drug resistance [5,6,7,8,9,10,11].

Despite not being a recent phenomenon, antimicrobial resistance (AMR) was noted to develop considerably in the last few decades, bacteria, fungi, and yeasts becoming resistant to many traditional and modern synthetic drugs [12,13]. It has been estimated that AMR is responsible for 33,000 deaths and poses an economic burden of 1.5 billion EUR per annum in Europe alone, thus, being among the most severe healthcare problems [14].

One of the main reasons behind the ineffectiveness of antibiotic treatments is represented by the formation of biofilms [6]. In these multicellular, surface-associated communities of microbes, bacteria are embedded within a matrix of extracellular polymeric substances (EPS) containing polysaccharides, extracellular DNA, and proteins [15,16,17]. Particularly, it was noted that the surface of biomaterials implanted into the human organism are prone to adherence and colonization with microorganisms, ultimately leading to the development of biofilms that protect the underlying bacteria from the host’s defense system and antibacterial substances [18,19,20,21].

Within this context, it is essential to create better alternatives in fighting recalcitrant biofilms. One attractive possibility is to take advantage of nanotechnology advances and translate them into developing novel antibiofilm bioactive surfaces. More specifically, coating medical devices and implants with an antimicrobial nanostructured layer represents a promising therapeutic option [22,23,24,25]. To obtain such bioactive coatings, the matrix-assisted pulsed laser evaporation (MAPLE) technique has recently attracted increasing scientific interest. MAPLE is a non-contact, contamination-free process; it offers high control over surface thickness and roughness, does not damage the structure, chemical properties, and functionality of the transferred material, and is considered an ideal technique for depositing hybrid organic-inorganic thin layers [26,27,28].

In this respect, considerable research has been directed to magnetic nanoparticles in general and magnetite in particular [29,30,31,32,33,34,35]. These magnetic iron oxide nanoparticles present a series of advantageous properties, such as availability, versatility, eco-friendliness, low cost, superparamagnetism, high saturation field, high electrical and thermal resistance, chemical stability, biocompatibility, biodegradability, non-toxicity to humans, intrinsic antimicrobial ability, and possibility for functionalization [36,37,38]. Therefore, magnetite nanoparticles (MNPs) are appealing for developing unconventional antimicrobials that can coat medical devices, transport drugs in a controlled and targeted manner, and minimize side effects due to the small amount of antibiotics they can deliver [36,39,40]. 

However, MNPs are not stable in air over a long period, having the tendency to oxidize to maghemite. Moreover, bare MNPs cannot form stable fluids and may easily agglomerate after production. To overcome these drawbacks, MNPs required in biomedical applications are usually protected by shells of different biocompatible materials, such as natural polysaccharides, inert synthetic materials, and organic acids with different structures [36,37,41,42].

Synthetic polymers have been widely applied in fabricating and coating nanoparticles for bioapplications [43]. Among them, one of the most commonly used is polyethylene glycol (PEG), which, despite not being bactericidal in its nature, has non-fouling properties that decrease the bacterial interaction with its surfaces. Furthermore, PEG enhances the biocompatibility of the core material, being also useful in the delivery of both antibiotics and alternative antibacterial drugs [5,19,44].

Polymyxins (PM) are a class of antibiotics that have been reported as efficient against many multidrug-resistant pathogens [45]. They generate a disruptive physicochemical effect, producing permeability changes in the outer membrane of bacteria. Through the developed transient “cracks”, a variety of molecules may pass, promoting the uptake of the perturbing peptide itself and resulting in cell death [46,47]. Nonetheless, as it happens with any antibiotic, the administration of high doses of polymyxin has led to the emergence of drug resistance, threatening the utility of this increasingly important last-line therapeutic option [45,48]. Therefore, synergistic therapies must be sought. 

In this regard, this study reports the combination of magnetite, PEG, and polymyxin-B into an antimicrobial approach. Particularly, we present the synthesis and characterization of functionalized MNPs and the fabrication of magnetite-based thin coatings. These new nanostructured coatings were characterized from the compositional, morphological, and biological points of view employing X-ray diffraction (XRD), thermogravimetric analysis with differential scanning calorimetry (TGA-DSC), scanning electron microscopy (SEM), transmission electron microscopy with selected area electron diffraction (TEM-SAED), Fourier-transform infrared spectroscopy (FT-IR), infrared microscopy (IRM), cell viability, and anti-biofilm tests.

## 2. Materials and Methods

### 2.1. Materials

Chemical substances used to synthesize the nanostructured materials, namely, ferrous sulfate (FeSO_4_), ferric chloride (FeCl_3_), ammonium hydroxide (NH_4_OH), polyethylene glycol (PEG), Dimethyl sulfoxide (DMSO), Polymyxin-B, chloroform (ACS grade), were purchased from Sigma Aldrich (Merck Group, Darmstadt, Germany). All chemicals were used without further purification, and all solutions were prepared using ultrapure water (MiliQ^®^, Merck Millipore, Burlington, MA, USA).

### 2.2. Preparation of Fe_3_O_4_@PEG and Fe_3_O_4_@PEG/PM

The MNPs functionalized with PEG were synthesized through a co-precipitation method, involving the prior preparation of two solutions. The first solution, containing the iron precursors, was prepared by adding 1.6 g of ferrous sulfate and 1 g of ferric chloride into 300 mL of demineralized water. The second solution contained 1 g of PEG400, 15 mL of ammonium hydroxide, and 300 mL of deionized water. The precursors’ solution was added dropwise to the alkaline solution under continuous stirring. The obtained solution was separated by means of a magnet; the product was washed and left to dry. After drying, it was ground in a mortar, resulting in 0.9 g of magnetite. The sample was split into two parts: one remained as it was, and the second one, was mechanically mixed with 0.05 g of polymyxin in 1 mL of chloroform. The mixing process was done when the chloroform was completely evaporated from the sample.

### 2.3. Preparation of Coatings Using MAPLE Technique

A ns-beam (*λ* = 248 nm, *τ*_FWHM_ = 25 ns, repetition rate = 15 Hz) from an KrF* excimer COMPexPro 205 Lambda Physics from Coherent was focused on Fe_3_O_4_@PEG and Fe_3_O_4_@PEG/PM samples (irradiation spot 30 mm^2^) generating laser fluences of 300, 400, and 500 mJ/cm^2^. For MAPLE deposition, the targets were prepared from powders mixed in DMSO (2%) and frozen at liquid nitrogen temperature. The coatings were deposited on 1 × 1 cm glass, voice prosthesis sections, and on double side polished (100) Si substrates, which were cleaned according to an internal procedure consisting of an ultrasonic bath with acetone, ethanol, and deionized water. The depositions were made for an average number of pulses of 47,000 at room temperature and 1 Pa residual gas. During the deposition, the target was continuously rotated to avoid deep crated formation, and the target to substrate distance was kept constant at 4 cm. 

### 2.4. Characterization Methods

#### 2.4.1. X-ray Diffraction (XRD)

An X-ray diffraction analysis of nanomaterial powders was performed with a Panalytical Empyrean diffractometer (step size 0.02, time per step 1 s) at room temperature. For all analyses made, Cu Ka radiations with l = 1.541874 Å were used. Samples were scanned at a Bragg 2Θ angle between 20–80. 

SEM was also used for the characterization of the obtained thin films.

#### 2.4.2. Thermogravimetric Analysis (TGA-DSC)

The thermal analysis TG-DSC for the precursors was performed with a Netzsch STA 449 C Jupiter apparatus. The samples (~20 mg) were placed in an open crucible made of alumina and heated with 10 K·min^−1^ from room temperature up to 900 °C, under the flow of 50 mL min^−1^ dried air. An empty alumina crucible was used as a reference.

#### 2.4.3. Transmission Electron Microscopy (TEM)

For the obtaining of TEM micrographs, a small amount of the sample powder was dispersed in pure ethanol and subjected to an ultrasonic treatment for 15 min. The sample was placed on a carbon-copper grid and left to dry at room temperature. For recording TEM micrographs, a Tecnai^TM^ G2 F30 S-TWIN high-resolution transmission electron microscope from FEI Company (Hillsboro, OR, USA) equipped with selected electron diffraction was operated in the transmission mode, at a 300 kV voltage, with point and line resolutions of 2 Å and 1 Å, respectively.

#### 2.4.4. Fourier Transform Infrared Spectroscopy (FTIR)

To investigate the integrity of functional groups characteristic to synthesized particles, a reduced quantity of particle suspension was analyzed using a Nicolet 6700 FTIR spectrometer from Thermo Fischer Scientific (Waltham, MA, USA). The measurements were performed at room temperature, 32 scans being collected in the range between 4000 and 1000 cm^−1^, with a 4 cm^−1^ resolution. The recording of the as-acquired information as possible by connecting the spectrometer to a unity of data processing using the Omnic Picta 8.2 software (Thermo Fischer Scientific, Waltham, MA, USA). 

#### 2.4.5. Infrared Microscopy (IRM)

IR mapping (IRM) was performed on a Nicolet iN10 MX FT-IR microscope with an MCT liquid nitrogen cooled detector in the 4000–800 cm^−1^ range. The spectral collection was made in reflection mode at 8 cm^−1^ resolution. A total of 32 scans were co-added and converted to absorbance for each spectrum using OmincPicta software (Thermo Scientific, Waltham, MA, USA). About 250 spectra were analyzed for each sample. The absorption peaks of C-H and C-O functional groups in PEG and C-H, C-O, and C=O groups in PM were chosen as specific spectral markers. 

#### 2.4.6. Scanning Electron Microscopy (SEM)

To investigate the morphology of coatings and thickness of thin coatings, the samples were placed in the analysis chamber of the scanning electron microscope from FEI (Hillsboro, OR, USA) electron microscope. The obtained images are produced by recording the resultant secondary electron beam with an energy of 30 keV.

### 2.5. Antibiofilm Evaluation at 24 and 48 h

The bacterial strains employed in the antibiofilm evaluation, namely, *Staphylococcus aureus* (ATCC^®^ 25923) and *Pseudomonas aeruginosa* (ATCC^®^ 27853), were obtained from the American Type Cell Collection (ATCC, Manassas, VA, USA).

To test the effect of the nanostructured surfaces on biofilm production, the samples were sterilized through UV exposure for 20 min on each side. Further, each fragment of sterile material was individually placed in a well of a 6-well plate, followed by the addition of 2 mL of nutritive broth and subsequent inoculation of 50 μL of bacterial suspensions corresponding to the 0.5 McFarland density (~1.5 × 10^8^ CFU (colony-forming units)/mL). The as-prepared 6-well plates were incubated at 37 °C for 24 h. After incubation, the materials were washed with PBS, and the culture medium was replaced with a fresh one to ensure biofilm development. The plates were incubated for 24 and 48 h, after which the specimens were washed with PBS and placed in a sterile tube containing 1 mL of PBS. The tubes were vigorously vortexed for 30 s to detach the cells from the biofilm. The as-obtained cell suspensions were diluted and seeded on plates with solidified culture medium to obtain and quantify the number of colony-forming units (CFU/mL).

### 2.6. Biocompatibility Assay

The biocompatibility of the synthesized materials was tested on murine 3T3-E1 osteoblasts. The cell culture was maintained at 37 ℃, in a humid atmosphere with 5% CO_2_, using Dulbecco’s Modified Eagle’s Medium (DMEM) supplemented with 10% fetal bovine serum. The samples were sterilized by UV exposure for 20 min on each side and placed in a 6-well plate, followed by the addition of cells at a density of 4 × 10^4^ cells/cm^2^. Cell visualization after 24 h of incubation was performed on an inverted phase-contrast microscope (Olympus IX71, Tokyo, Japan).

An MTT (3-(4,5-dimethylthiazol-2-yl)-2,5-diphenyltetrazolium bromide) test was employed for assessing the viability of cells in the presence of the analyzed materials. This biocompatibility assay works on the principle of reducing yellow MTT salt to dark blue formazan crystals by metabolically active cells. The reducing coefficient is proportional to the number of viable cells, thus indicating cellular integrity. After medium removal, the cells were washed with PBS, MTT solution (1 mg/mL) was added, and the cultures were incubated at 37 ℃ for two hours in the dark. The MTT solution was discharged and replaced with an equal volume of isopropanol to resuspend the formazan crystals through pipetting until their complete solubilization. The spectrophotometric measurement of absorbance was performed at a wavelength of 595 nm using an Appliskan Thermo Scientific plate reader. 

The nitric oxide (NO) concentration released from cells into the cell culture medium was determined using the Griess method. A volume of 80 μL from each culture media collected after treatment was pipetted in 96-well plates. A solution of 1% sulfanilamide (Sigma-Aldrich, Darmstadt, Germany) and 0.1% *N*-(1-naphthyl)-ethylenediamine dihydrochloride (Sigma-Aldrich) was freshly prepared right before being added to media in the wells in a 1:1 (*v*/*v*) ratio. The absorbance of the formed colored product was spectrophotometrically measured at 550 nm using an Appliskan Thermo Scientific plate reader.

### 2.7. Statistical Analysis of Data

Microbiological and biocompatibility results were performed in triplicates (n = 3) and analyzed using GraphPadIn Stat and Prism software, by applying One-way Analysis of Variance (ANOVA) test. Statistically significant data were considered for a value of p less than 0.05.

## 3. Results

### 3.1. Characterization of Nanoparticles

**Figure 1:**
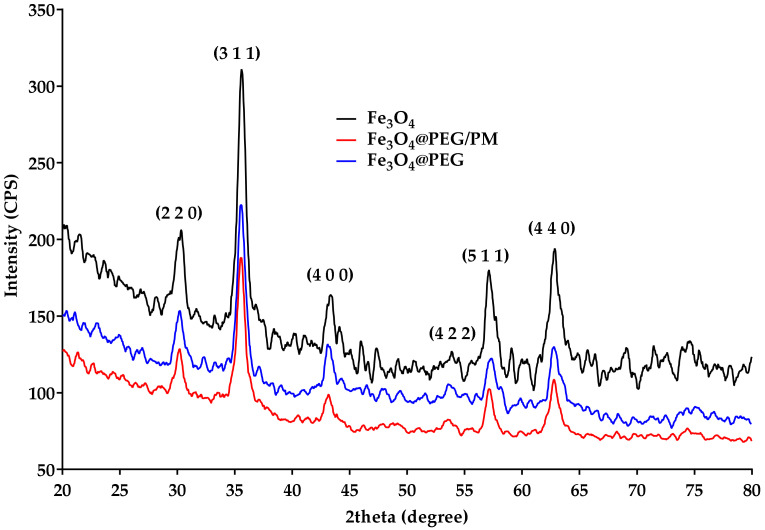
X-ray diffractogram of Fe_3_O_4_, Fe_3_O_4_@PEG, and Fe_3_O_4_@PEG/PM.

The XRD patterns of the bare and surface-modified magnetite particles were overlapped in Figure 1. According to specialty data, the common strong diffraction peaks correspond to the (111), (220), (311), (400), (422), (511), and (440) diffraction planes of the crystallographic system. As it can be observed, the analyzed sample presents high crystallinity. Moreover, all the peaks agree with the standard spectrum of Fe_3_O_4_ with a cubic inverse spinel structure (DB card No. 9006242).

**Figure 2:**
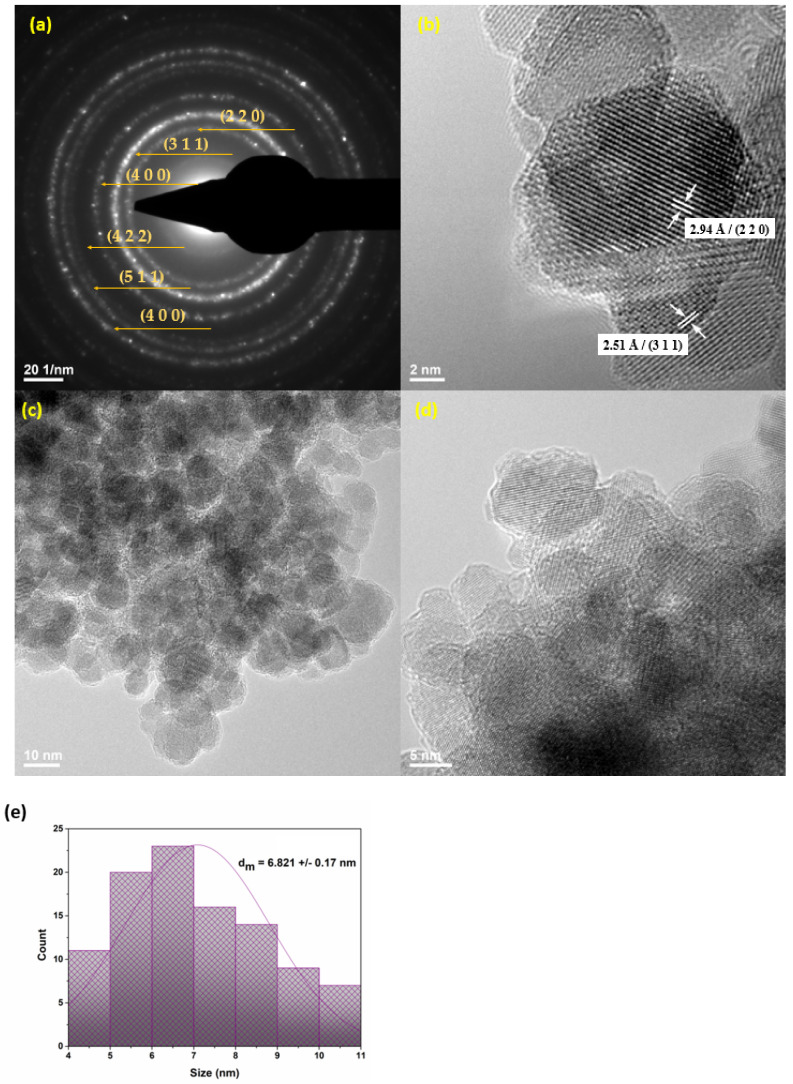
SAED pattern (**a**), HR-TEM image (**b**), and TEM images (**c**,**d**) of Fe_3_O_4_@PEG/PM; (**e**) size distribution.

TEM and SAED analyses were used to retrieve information regarding the structure of MNPs. Through the acquired TEM images (Figure 2b–d), the morphology of the obtained nanoparticles and growth direction can be emphasized. From a structural point of view, the particles have a quasi-spherical shape, their agglomeration leading to cluster formation. At higher magnification (Figure 2b), it was established that the nanoparticles have dimensions of ~6.8 nm. The SAED diffraction pattern visible in Figure 2 highlights the complex nature of the ring, proving the crystallinity of the material. The formed planes, namely (111), (220), (311), (400), (422), (511), and (440), are in agreement with both literature [49] and previously XRD-obtained data, confirming that PEG-functionalized magnetite is a polycrystalline material.

**Figure 3:**
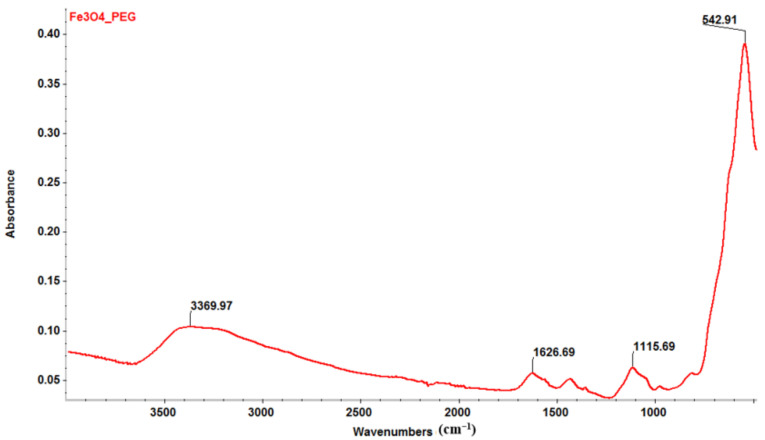
FT-IR spectrum of Fe_3_O_4_@PEG/PM nanoparticles.

Experimental evidence supporting the formation of a thin PEG layer on the surface of MNPs can be seen in Figure 2d. Also, the FT-IR spectrum (Figure 3), highlights the presence of PEG. A first broad absorption band can be noticed between 3500 and 3000 cm^−1^, with a maximum of around 3369.97 cm^−1^ is assigned to the OH group characteristic to the PEG and PM. Between 1650 and 1600 cm^−1^, with the maximum around 1626.69 cm^−1^, the absorption band can be attributed to C=O characteristic to PM. At ~1115.60 cm^−1^, there can be noticed another absorption band corresponding to C-O vibrations characteristic to PEG. Also, the Fe-O absorption band was identified at 542 cm^−1^, characteristic of magnetite.

**Figure 4:**
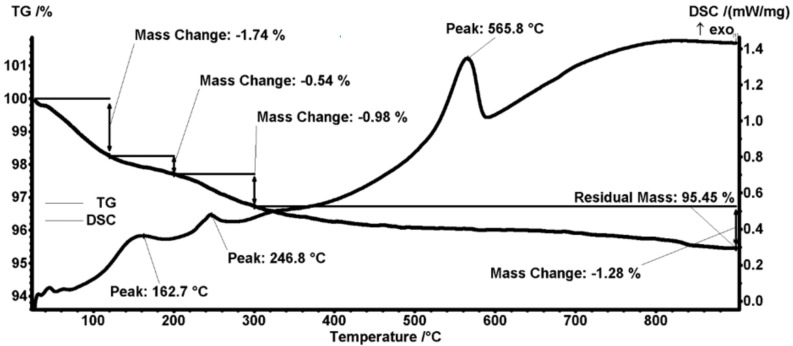
Thermogravimetric analysis of Fe_3_O_4_ nanoparticles.

The thermal analysis of the pristine magnetite particles (Figure 4) indicates the presence of four mass loss processes. The first mass loss between RT-120 °C represents 1.74% and is due to eliminating water molecules, accompanied by a slight endothermic effect on the DSC curve. Two other successive mass losses are registered between 120–200 °C and between 200–300 °C, accounting for 0.54% and 0.98%, respectively; both of these mass changes are accompanied by exothermic effects and are attributed to the oxidation Fe^2+^ to Fe^3+^ (transformation of magnetite to maghemite) and oxidation of organic impurities found on the surface of Fe_3_O_4_ nanoparticles [50]. The mass loss continues slowly after 300 °C, ultimately reaching a residual mass of 95.45%. Within this final mass-loss process, an exothermic effect is noticed, with a strong peak at 565.8 °C caused by the transformation of maghemite into hematite [51].

**Figure 5:**
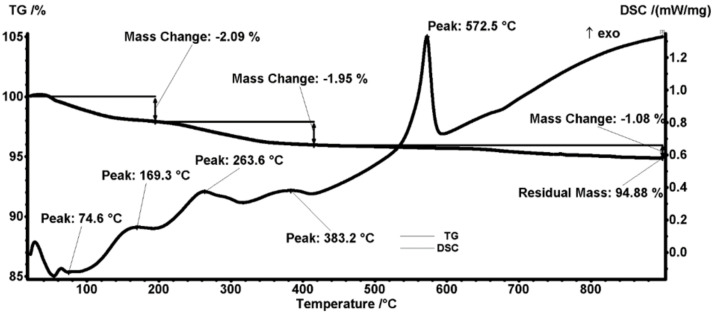
Thermogravimetric analysis of Fe_3_O_4_@PEG nanoparticles.

The thermal analysis for the Fe_3_O_4_@PEG nanoparticles (Figure 5) shows three distinct mass loss stages. The first one occurs in the interval RT-200 °C when 2.09% of the initial mass is lost. The process can be attributed to the water elimination from the sample, as an endothermic effect accompanies it. The exothermic effect from 169.3 °C can be attributed to the transformation of magnetite to maghemite (oxidation of Fe^2+^ to Fe^3+^) [52]. The second mass loss process, between 200–420 °C, accounts for 1.95% and is the main process of degrading the organic part by oxidation. Two exothermic effects, with peaks at 263.6 and 383.2 °C, accompany the oxidative degradation of PEG. The sample continues to slowly lose mass, 1.08%, up to 900 °C, while the residual mass is noted to be 94.88%. The exothermic peak registered at a temperature of 572.5 °C corresponds to the physical transformation of maghemite into hematite [53].

By comparing the mass loss values for both samples, we estimate that the organic compound deposited on magnetite particles is present in an amount of ~0.59 g per 100 g of Fe_3_O_4_@PEG composite.

### 3.2. Thin Coatings Characterization

**Figure 6:**
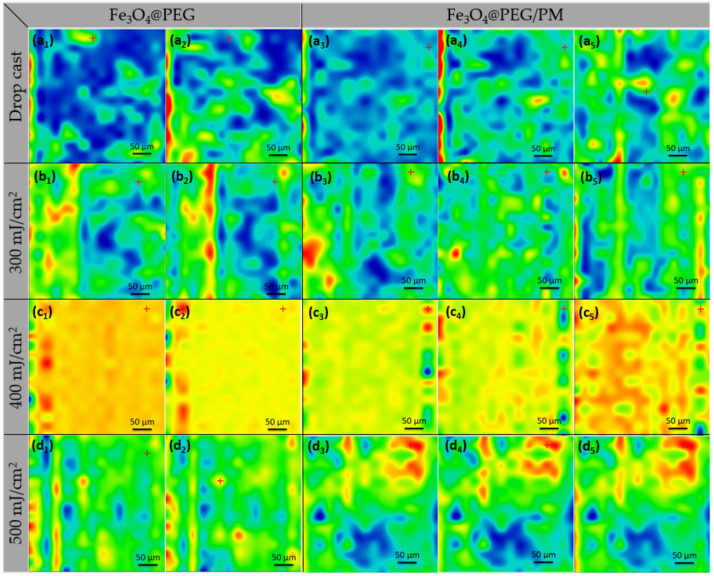
IR maps of Fe_3_O_4_@PEG (1,2) and Fe_3_O_4_@PEG/PM (3,4,5); dropcast (**a**), F = 300 mJ/cm^2^ (**b**), F = 400 mJ/cm^2^ (**c**), F = 500 mJ/cm^2^ (**d**), based on the distribution of C-H (1), C-O (2), C-H (3), C-O (4) and (C=O (5), assigned to PEG (1,2,3,4) and PM (5).

**Figure 7:**
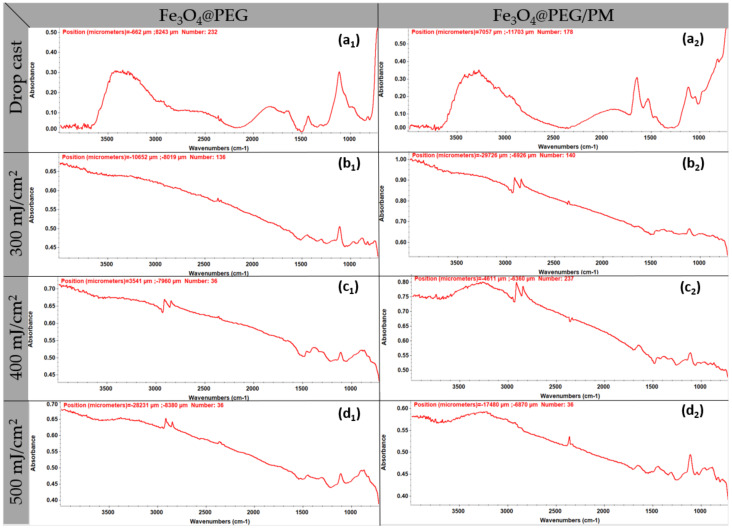
IR spectra of Fe_3_O_4_@PEG (1) and Fe_3_O_4_@PEG/PM (2): dropcast (**a**), F = 300 mJ/cm^2^ (**b**), F = 400 mJ/cm^2^ (**c**), F = 500 mJ/cm^2^ (**d**).

Relevant compositional data were obtained by IR-mapping and IR-spectra (Figure 6 and Figure 7). Figure 6 presents the results related to the IR-mapping of dropcast (a) and thin coatings obtained at different laser fluence: F = 300 mJ/cm^2^ (b), F = 400 mJ/cm^2^ (c), F = 500 mJ/cm^2^ (d). 

By analyzing the dropcast IR maps (Figure 6a), it can be seen that the samples are not uniformly deposited. Dropcast was used as a control to highlight the optimum laser fluence better. This was monitored by a color gradient of the composition with absorbance maximum (red) and minimum (blue) for several characteristic functional groups: C-H and C-O (characteristic for PEG) were monitored for Fe_3_O_4_@PEG and C-H, C-O (characteristic for PEG), and C=O (characteristic for PM) was monitored for Fe_3_O_4_@PEG/PM.

For F = 300 mJ/cm^2^, IR maps highlight that the intensity of the absorption band varies from low to medium and even high in some areas. It can also be seen that the sample was not uniformly deposited (may blue zones).

For F = 400 mJ/cm^2^, IR maps highlight that, in the case of Fe_3_O_4_@PEG/PM, the intensity of the absorption band varies from medium (yellow-orange) to medium-high (orange-red) in the scanned surface area. It can also be seen that there is a very low tendency of agglomeration of the nanostructures (some isolated red area).

For F = 500 mJ/cm^2^, IR maps highlight that the intensity of the absorption band varies, and it can be seen that the sample was not uniformly deposited (in all cases).

Thus, at the lowest laser fluence, the deposited amount is not enough to give a uniform layer, while, at the highest fluence, there is a high tendency of agglomeration.

Similar results were obtained for both samples, Fe_3_O_4_@PEG and Fe_3_O_4_@PEG/PM.

Supporting complementary information was obtained by analyzing IR spectra of Fe_3_O_4_@PEG and Fe_3_O_4_@PEG/PM dropcast and coatings produced at laser fluences of 300, 400, and 500 mJ/cm^2^ (Figure 7).

By comparing these spectra, it can be observed that the F = 200 mJ/cm^2^ presents a low absorbance intensity due to a non-homogenous coating and insufficient amount of transferred material, while F = 300 mJ/cm^2^ and F = 400 mJ/cm^2^ present a medium-high absorbance intensity. Identified peaks are characteristics of PEG and PM.

**Figure 8:**
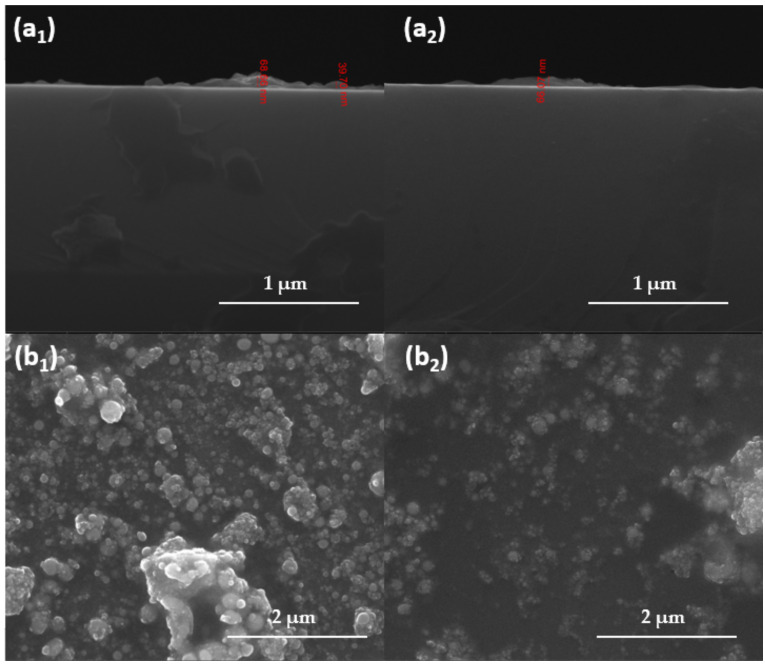
SEM images of Fe_3_O_4_@PEG (1) and Fe_3_O_4_@PEG/PM (2): (**a**) cross-section; (**b**) coatings surface.

Coatings were further characterized by SEM. The micrographs are plotted in Figure 8 and offer information regarding the textured aspect of the coating formed at a laser fluence of 400 mJ/cm^2^ (Figure 8a), while the images from Figure 8b provide useful data concerning the thickness of the nanostructured coatings obtained at a laser fluence of 400 mJ/cm^2^. As it can be seen, the thickness of both coatings varies between 40 and 70 nm. The micrographs show a continuous material area with a wave-like cross-section aspect, which delineates the small agglomeration tendency.

About the surface, it can be seen that the quasi-spherical shape of the nanoparticles is not altered during MAPLE processing. There is a small tendency of agglomeration, and it can be seen that magnetite nanoparticles are embedded in a PEG matrix.

As the results obtained by IRM analysis underlined that selecting a laser fluence of 400 mJ/cm^2^ is optimum for producing thin magnetite layers, the voice prosthesis sections coated at this fluence were subjected to in vitro evaluations. 

**Figure 9:**
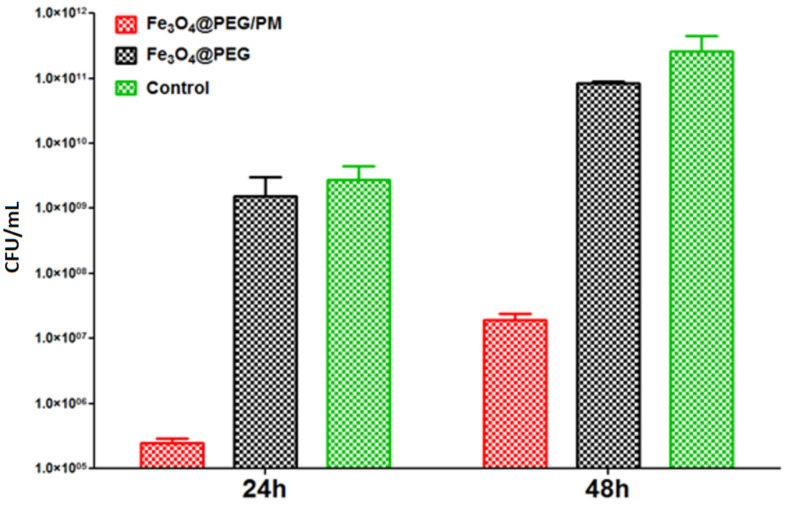
Evaluation of biofilm development after 24 and 48 h of incubation in the presence and absence of PEG-based thin film for *S. aureus* strain.

The evaluation of the anti-adherent properties of the modified voice prosthesis fragments against *S. aureus* biofilm led to favorable results (Figure 9). At 24 and 48 h after incubation, the control cell cultures present a high level of CFU/mL, 1.3 × 10^9^ CFU/mL and 1.4 × 10^11^ CFU/mL, respectively. In comparison, the nanostructured surface of modified voice prosthesis sections reduced the number of CFU/mL up to four orders of magnitude (showing 1.4 × 10^5^ CFU/mL for 24 h biofilms and 1.1 × 10^7^ CFU/mL in 48 h biofilms, in the case of the Fe_3_O_4_@PEG/PM coating). These results demonstrate a strong anti-adherent character against *S. aureus* biofilm development. 

**Figure 10:**
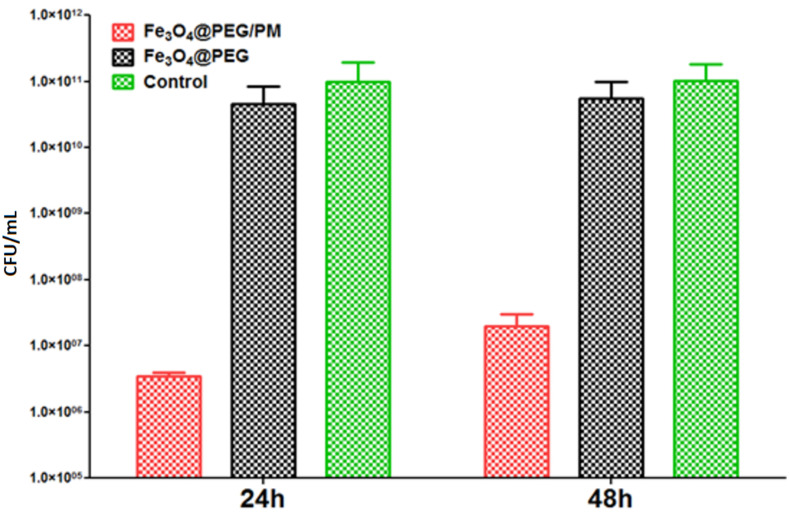
Evaluation of biofilm development after 24 and 48 h of incubation in the presence and absence of PEG-based thin film for *P. aeruginosa* strain.

In the case of *P. aeruginosa* tests (Figure 10), the control CFU/mL values were similar for 24 and 48 h, namely ~10^11^ CFU/mL. These results demonstrate the high affinity of *P. aeruginosa* strains for colonizing the surfaces of medical devices, a reason for which this opportunistic pathogen is associated with numerous nosocomial, difficult-to-treat infections. Moreover, compared to the other analyzed microorganism, a lower inhibitory effect is noticed for both nanocomposites. Specifically, for Fe_3_O_4_@PEG, the CFU/mL values are reduced only by one fold, up to 1.7 × 10^10^ CFU/mL after 24 h, and 1.8 × 10^10^ CFU/mL after 48 h. However, for the Fe_3_O_4_@PEG/PM nanostructured coating, a more significant inhibitory effect is noticed; this material was able to reduce the colonization of *P. aeruginosa* up to five-folds, down to 1.5 × 10^6^ CFU/mL for 24 h biofilms and 1.2 × 10^7^CFU/mL after 48 h of incubation. 

**Figure 11:**
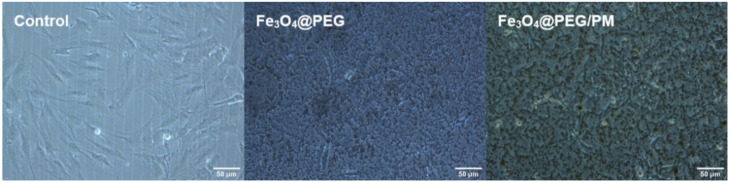
Morphology of MC3T3-E1 osteoblasts cultivated in the absence (control) and presence of Fe_3_O_4_@PEG and Fe_3_O_4_@PEG/PM samples.

The optical micrographs are shown in Figure 11 display the murine MC3T3-E1 osteoblasts after 24 h of incubation with PEG-based thin film. The specific aspect of osteoblasts can be noticed both for the control and the investigated coatings. It can be noticed that the cells attached and elongated on the modified surface.

**Figure 12:**
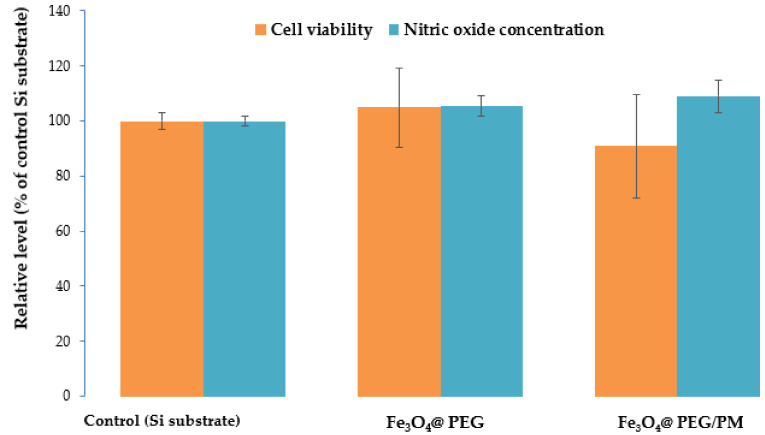
Cell viability and nitric oxide concentration after 24 h of incubation of MC3T3-E1 osteoblasts with Fe_3_O_4_@PEG and Fe_3_O_4_@PEG/PM samples. The measurements were performed in triplicate (n = 3) and the results were presented as average ± standard deviation relative to the control.

The Fe_3_O_4_@PEG samples did not induce significant changes in the number of viable cells and nitric oxide level compared to the control (Figure 12), confirming its biocompatibility. Also, the Fe_3_O_4_@PEG/PM sample decreased the viability of osteoblasts by only 9% of control, which was in agreement with the 9% increase of nitric oxide release. This small percentage confirmed the good biocompatibility of this Fe_3_O_4_@PEG/PM system. It can be suggested that polymyxin toxicity was reduced in the presence of PEG-functionalized MNPs.

## 4. Conclusions

MNPs coated with PEG and polymyxin-B have been successfully synthesized by the co-precipitation method and were characterized through various techniques. The dimensions between 5 and 9 nm, the high crystallinity of the products, their enhanced biocompatibility, reduced toxicity, and inhibitory effects against biofilm formation render these coatings useful in antimicrobial therapy. In particular, the coatings produced by the MAPLE technique at 400 mJ/cm^2^ laser fluence proved the most uniform deposition and stoichiometric transferred, among the tested laser fluences. Their further biological investigations led to favorable results that recommend these materials for developing new surfaces or biomedical devices (i.e., voice prosthesis) that prevent biofilm infections.

## Data Availability

Available from authors, upon request.
